# A sensitive, robust method for determining natural and synthetic hormones in surface and wastewaters by continuous solid-phase extraction–gas chromatography–mass spectrometry

**DOI:** 10.1007/s11356-022-19577-1

**Published:** 2022-03-15

**Authors:** Safae Chafi, Evaristo Ballesteros

**Affiliations:** Department of Physical and Analytical Chemistry, E.P.S of Linares, University of Jaén, Avenida de La Universidad, 23700 Linares, Jaén Spain

**Keywords:** Natural and synthetic hormones, Water, Continuous solid-phase extraction, Microwave-assisted derivatization, Gas chromatography–mass spectrometry

## Abstract

Over recent decades, steroidal estrogens have become an emerging and very serious issue as they pose a serious threat to living organisms, soil, plants, and water resources in general. Estrogens have therefore been the subject of considerable scientific attention in order to develop new methodologies for its determination, being able of detecting them at very low concentrations. Those procedures minimize or eliminate the consumption of organic solvents and reagents that may be incompatible with the environment. In this respect, we developed a sensitive, selective method for the simultaneous determination of thirteen natural and synthetic hormones present at the nanogram-per-liter level in various types of water by using continuous solid-phase extraction in combination with gas chromatography and mass spectrometry (GC–MS). The target analytes were preferentially sorbed on an Oasis HLB sorbent column (80 mg) and eluted with acetone (600 µL) for derivatization with a mixture of 70 µL of *N*,*O*-*bis*(trimethylsilyl) trifluoroacetamide and trimethylchlorosilane and 35 µL of petroleum ether in a household microwave oven at 200 W for 4 min. Under optimum conditions, the ensuing method exhibited good linearity (*r* ≥ 0.998), good precision (RSD ≤ 7%), high recoveries (92–103%), and low detection limits (0.01–0.3 ng L^−1^). The method outperforms existing alternatives in robustness, sensitivity, throughput, flexibility—it allows both estrogens, progestogens, and androgens to be determined simultaneously—and compliance with the principles of Green Chemistry. It was successfully used to analyze various types of water samples (mineral, tap, well, pond, swimming pool, river, and waste) that were found to contain four estrogens (estrone, 17β-estradiol, 17α-ethinylestradiol, and hexestrol), two progestogens (testosterone, dihydrotestosterone), and one progestogen (progesterone) at concentrations ranging from 3.0 to 110 ng L^−1^.

## Introduction

Human health and well-being are closely linked to environmental quality. The growing population urbanization and modernization in many countries have raised serious environmental problems. In recent years, the number of micropollutants released to the environment has increased massively. This has been especially so with endocrine disrupting chemicals (EDCs) reaching aquatic ecosystems, which have received growing attention from the international scientific community owing to their strong potential impacts on human and ecosystem health. According to a scientific statement of the Endocrine Society, EDCs can have deleterious effects on male and female reproduction, breast development and health, thyroid metabolism, weight, and prostate, neuroendocrine, and cardiovascular functions (Diamanti-Kandarakis et al. 2009). Natural and synthetic hormones such as estrogens, progestogens, and androgens, which are among the most important biologically active EDCs, are produced naturally in the mammal body or synthetically created for use in birth control pills, growth promoters for livestock, and medical applications. Hormones can reach the aquatic environment through direct discharge, incomplete removal in wastewater treatment plants or agricultural runoff (Adeel et al. 2017). Some studies have revealed that steroid hormones can have adverse effects on aquatic organisms (e.g., fish feminization, infertility, and the development of physical abnormalities) at concentrations similar to those found in the aquatic environment (a few nanograms per liter; Sumpter and Jobling 2013; Fent 2015).

Natural and synthetic hormones have been encountered in various types of aquatic samples including surface, ground, drinking, and wastewater (Fent 2015; Golovko et al. 2018; Shen et al. 2018; Zhang and Fent 2018).Also, the American Environmental Protection Agency (US EPA) has included five (norethindrone, estrone, 17β-estradiol, 17α-ethinylestradiol, and estriol) on their Final Fourth Contaminant Candidate Lists US EPA (EPA 2016), and three estrogens (17α-ethinylestradiol, 17β-estradiol, and estrone) were recently added to the second “watch list” of substances for EU-wide monitoring in the field of water policy proposed by the European Union in Decision 2018/840/EU (EC 2018, 2020). However, detecting and monitoring of other classes of natural and synthetic hormones such as progestogens and androgens remains difficult and their environmental risks poorly known as a result. This has raised the need to acquire high-quality data by monitoring hormone concentrations in the environment and led to these compounds being classified in a new group of EDCs of concern for environmental regulations worldwide (Bergman et al. 2013).

Developing sensitive, selective analytical methods for determining hormones in environmental water samples is made difficult by the typical complexity of samples resulting in matrix interferences and the usually very low analyte concentrations (subnanogram-per-liter level) in aquatic matrices hindering their measurement. This shortcoming can be circumvented by using various analytical procedures including preconcentration and clean-up by liquid–liquid extraction (LLE; Fredj et al. 2015) or solid-phase extraction (SPE; Suri et al. 2012; Vega-Morales et al. 2012; Caban et al. 2015; Huysman et al. 2017; Golovko et al. 2018; Shen et al. 2018; Zhang and Fent 2018). SPE is an effective choice for preconcentrating analytes and increasing the sensitivity of analytical methods as a result. SPE sorbents are chosen according to the properties of the target analytes and the nature of the matrix. The most widely used sorbent for hormones is Oasis HLB, a hydrophilic–lipophilic copolymer of *N*-vinylpyrrolidone and divinylbenzene (Azzouz et al. 2010; Migowska et al. 2012; Vega-Morales et al. 2012; Kumirska et al. 2015; Shen et al. 2018; Zhang and Fent 2018) that efficiently retains both polar and nonpolar compounds. Some authors, however, have used mesoporous silica functionalized with octadecyl groups (SBA-15-C18; Gañán et al. 2015) or the macroporous neutral sorbent IonPac NG1 (Guo et al. 2013) to extract natural and synthetic hormones from water. Also, other sample preparation techniques such solid-phase microextraction (Aftafa et al. 2014), stir-bar sorptive extraction (Xu et al. 2014; Almeida and Nogueira 2015), and dispersive liquid–liquid microextraction (Chang and Huang 2010; Martín et al. 2015) have been used to determine hormones in water samples.

Chromatographies are the most widely used instrumental techniques for the simultaneous determination of steroid hormones in environmental water samples. Coupling gas chromatography (GC) or liquid chromatography (LC) to single (MS) or tandem mass spectrometry (MS/MS) provide good separation, sensitivity, and limits of detection. The LC–MS and LC–MS/MS combinations are especially frequently used because they enable direct analysis without derivatization of the analytes (Vega-Morales et al. 2012; Guo et al. 2013; Aftafa et al. 2014; Martín et al. 2015; Huysman et al. 2017; Golovko et al. 2018; Shen et al. 2018). However, LC–MS/MS was found to be subject to stronger matrix interferences—and hence to exhibit reduced signal-to-noise ratios, stability, and accuracy—than GC–MS and GC–MS/MS (Grover et al. 2009). GC–MS equipment is economical to purchase and maintain, operationally simple, environmentally friendly due to the inert gases of the mobile phase, and provides excellent chromatographic resolution. Another advantage of GC is that capillary columns are very long and have hundreds of thousands of theoretical plates more than LC columns, which makes GC a good performance for the separation of a large number of hormones. A drawback of the LC–MS technique is that the ionization efficiency is low for some hormones, which would block their detection by LC–MS (Robles et al. 2017; Ben Sghaier et al. 2017). Therefore, LC-MS is attractive tool for identifying and quantifying natural and synthetic hormones in environmental matrices (Migowska et al. 2012; Suri et al. 2012; Albero et al. 2013; Caban et al. 2015; Kumirska et al. 2015; Ben Sghaier et al. 2017). The GC–MS technique requires derivatizing the analytes in order to increase their volatility and thermal stability for improved chromatographic separation and sensitive, selective detection. Hormones are usually derivatized by silylating their hydroxyl groups with *N*,*O*-*bis*(trimethylsilyl) trifluoroacetamide (BSTFA) (Hernando et al. 2004; Ben Sghaier et al. 2017) or *N*-methyl-*N*(trimethylsilyl) trifluoroacetamide (MSTFA) (Huang et al. 2015).

The primary aims of this work were *(a)* to develop a robust, fast, and sensitive analytical method that is capable of simultaneously determining natural and synthetic estrogens progestogens and androgens in environmental water samples at very low concentrations, at which they could affect both the living beings that inhabit them and the human beings that consume these waters; *(b)* to select a procedure that is compatible with the aims of in which the expenditure of samples, reagents, and solvents is kept to a minimum; *(c)* to optimize the experimental procedure for efficient automated solid-phase extraction (SPE), complete, expeditious derivatization with a microwave oven, and accurate quantification by gas chromatography–mass spectrometry (GC–MS); *(d)* to use the proposed method for the analysis of environmental samples in order to confirm the presence of natural and synthetic hormones in various types of water samples (tap, well, pond, swimming pool, river, wastewater, and bottled) collected in Spain and Morocco; and *(e)* to compare the results obtained in the analysis of the samples with those obtained by other researchers in studies carried out around the world.

## Materials and methods

### Chemicals and reagents

All analytical standards used were of the highest commercially available purity. The hormones (17β-estradiol, estrone, 17α-ethinylestradiol, estriol, hexestrol, diethylstilbestrol, progesterone, 19-norethindrone, levonorgestrel, testosterone, dihydrotestosterone, androstenedione, and pregnenolone) were all purchased from Sigma–Aldrich (Madrid, Spain) in the highest available purity. Chromatographic-grade solvents (acetone, methanol, ethanol, *n*-hexane, petroleum ether, acetonitrile, and ethyl acetate), the derivatizing reagents (*N*,*O*-*bis*(trimethylsilyl) trifluoroacetamide (BSTFA) and trimethylchlorosilane (TMCS)), and triphenylphosphate were also purchased from Sigma–Aldrich. Hydrochloric acid (reagent-grade, 37% HCl), potassium chloride (KCl), and sodium hydroxide (NaOH) were obtained from Fluka (Madrid, Spain). Amberlite XAD-2 (particle size 20–60 µm) and reversed phase silica with octadecyl functional groups (RP-C18, particle size 40–63 µm), Isolute NH_2_ (aminopropyl, particle size 45–65 µm), Florisil (particle size 16–30 µm), and silica gel (particle size 15–35 µm) were equally obtained from Sigma–Aldrich. Oasis HLB (particle size 50–65 µm) and LiChrolut EN (particle size 40–120 µm) were supplied by Waters (Madrid, Spain) and Merck (Madrid, Spain), respectively.

Individual stock standards solutions of the target analytes at a concentration of 5 g L^−1^ were prepared in methanol—by exception, those of estrone and levonorgestrel were prepared in acetone in order to avoid crystallization. All standards solutions were stored in amber glass-stoppered bottles at 4 °C. Also, all aqueous standards were prepared in ultrapure water from a Milli-Q apparatus (Millipore, Bedford, MA, USA).

### Equipment

Hormones were identified and quantified by using a Focus gas chromatograph coupled to a DSQ II mass spectrometer equipped with an AI/AS 3000 autosampler and controlled by a computer running Xcalibur software (Thermo Electron SA, Madrid, Spain). The instrument was equipped with a DB-5 fused silica capillary column (30 m, 0.25 mm i.d., 0.25 µm) coated with 5% phenylmethyl polysiloxane (Supelco, Madrid, Spain) for chromatographic separation with helium (purity 6.0) at a constant flow rate of 1 mL min^−1^ as the carrier gas. The temperature program was as follows: 130 °C for 0.5 min, 40 °C min^−1^ ramp to 240 °C, ramp to 280 °C at 5 °C min^−1^, and holding for 3.75 min. The total analysis time for a GC run was 15 min. The injector was operated in the splitless mode at a set temperature of 280 °C. The transfer line and ion source temperature were 280 and 200 °C, respectively, and the time for solvent delay set at 5 min. The mass range from 60 to 500 amu was used for full scan analysis. Data were acquired in the selected ion monitoring mode, using an ionization energy of 70 eV. The *m*/*z* values for each analyte are listed in Table 1. Quantitation was based on peak areas relative to the internal standard (IS).

**Table 1:** Retention time, pk_a_, logK_o/w_, analytical figures of merit and mass values used to the determination of hormones in water samples by SPE-GC–MS

Compounds	pK_a_	Log K_o/w_	Retention time (min)	Linear range (ng L^−1^)	Correlation coefficient	LOD (ng L^−1^)^b^	Precision RSD (%)^c^		*m/z*^d^		
							Within-day	Between-day	[M]^+^	[M-15]^+^	Additional ions
Estrogens											
Hexestrol	9.90	5.10	7.91	0.04–800	0.9997	0.01	4.6	6.8	414	399	**207**, 179
Diethylstilbestrol	9.13	5.10	7.99	0.40–800	0.9998	0.10	3.0	4.9	**412**	397	383, 217
Estrone	10.20	3.69	11.05	0.04–800	0.9993	0.01	4.2	6.8	**342**	327	218, 257
17β-estradiol	10.27	4.13	11.31	0.04–800	0.9982	0.01	3.5	7.0	**416**	401	285, 326
17α-ethinylestradiol	10.24	4.25	12.62	0.04–800	0.9992	0.01	5.3	6.8	440	**425**	232, 300
Estriol	10.25	2.52	13.73	0.04–800	0.9992	0.01	5.5	7.0	**504**	489	147, 311
Androgens											
Testosterone	15.06	3.17	11.65	0.50–800	0.9998	0.15	3.9	6.2	**360**	345	270, 226
Dihydrotestosterone	n.a.^a^	3.55	10.90	0.50–800	0.9992	0.15	4.1	5.9	362	347	**129**, 272
Androstenedione	n.a.^a^	2.71	11.44	1.00–800	0.9996	0.30	3.9	6.1	**286**^**e**^	–	244, 148
Progestogens											
Progesterone	n.a.^a^	3.80	13.60	0.18–800	0.9981	0.05	3.7	4.5	314^e^	–	**124**, 272
Norethindrone	13.09	2.99	11.60	1.00–800	0.9997	0.30	5.5	6.7	370	355	**231**, 298
Levonorgestrel	13.09	3.36	12.88	0.18–800	0.9998	0.05	4.1	4.6	384	369	**355**, 281
Others											
Pregnenolone	n.a.^a^	4.22	12.35	0.35–800	0.9995	0.10	4.0	4.7	388	373	**129,** 298

^a^Not available

^b^Limit of detection

^c^Relative standard deviation (n = 12) for ultrapure water spiked with 5 ng L^‒1^

^d^Base peaks used for quantification are boldfaced, [M]^+.^: ionized mass, [M-15]^+^: loss of a CH_3_ radical from the Si(CH_3_)_3_ group, *m/z* for IS (triphenylphosphate): 77, 170, 325, **326**

^e^Progesterone and androstenedione are determined as non-derivatized

The continuous solid-phase extraction system consisted of a Gilson Minipuls-3 peristaltic pump (Villiers-le-Bel, France) fitted with poly(vinyl chloride) tubes, two Rheodyne 5041 injection valves (Cotati, CA, USA) and a laboratory-made PTFE sorption column (5 cm, 3 mm id) containing 80 mg of Oasis HLB sorbent. The sorbent column, which was conditioned with 1 mL of methanol, 1 mL of acetone, and 2 mL of purified water, remained useful for at least 2 months.

### Sampling

Water samples (tap, mineral, well, pond, swimming pool, river, and waste) were collected in precleaned 500-mL amber glass bottles at various locations in Spain and Morocco, stored refrigerated at 4 °C, and analyzed within 3 days after collection in all instances. Bottled mineral water samples of the most popular brands were purchased locally. Suspended materials in the samples, which could block the SPE column, were removed by passage through Millipore-mixed cellulose ester membrane filters of 0.45 µm pore size. All samples were adjusted to neutral pH by adding dilute HCl or NaOH as required.

### Sample treatment

**Fig. 1:**
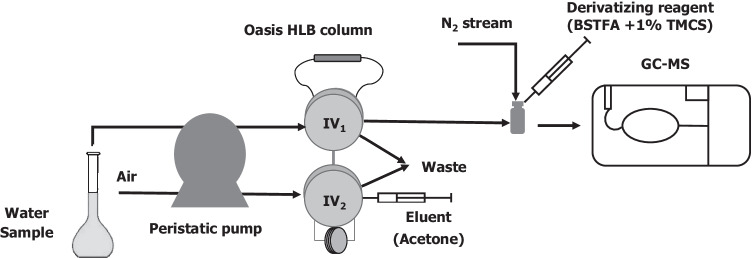
Continuous flow unit for the SPE extraction of hormones in water and their offline determination by gas chromatography. IV, injection valve; GC–MS, gas chromatograph–mass spectrometer

Samples were cleaned up and the target analytes preconcentrated by continuous solid-phase extraction SPE (Fig. 1). A volume of 100 mL of sample or standard solution at pH 7 adjusted with 0.1 M HCl or 0.1 M NaOH and containing a 0.04–800 ng L^−1^ concentration of each compound was passed at 5 mL min^−1^ through the sorbent column (80 mg of Oasis HLB located in the loop of injection valve 1 ( IV_1_)) to have natural and synthetic hormones immediately adsorbed and the sample matrix sent to waste. Then, the column was dried by passing an air stream at 5 mL min^−1^ before the loop of injection valve 2 (IV_2_) was filled with 600 µL of eluent (acetone containing a 500 ng L^−1^ concentration of triphenylphosphate as internal standard to avoid potential errors in measuring the final extract). Next, the column was eluted in the opposite direction of sample feeding. The organic extract was transferred into an air-tight 0.5-mL conical glass insert and evaporated to dryness under a gentle nitrogen stream. Samples should be thoroughly dried before derivatization in order to prevent the silylation reagents from interacting with moisture. The resulting dry residue was reconstituted with 70 µL of a mixture of BSTFA + 1% TMCS (derivatizing reagent) and 35 µL of petroleum ether. Then, the vials were closed and placed in a household microwave oven at 200 W for 4 min. After derivatization, 1 µL aliquots were injected into the GC–MS system for analysis.

## Results and discussion

### Derivatization

Derivatization is the most common and flexible choice for optimal chromatographic separation and MS measurement of analytes. The high polarity and thermal instability, and low molecular weight, of hormones can result in their easy adsorption on the chromatographic column or their decomposition at the injector port. This requires derivatizing them to increase their volatility and decrease their polarity—and hence dipole–dipole interactions—prior to injection into GC–MS for their determination at trace (subnanogram-per-liter) levels, something that can be easily and rapidly accomplished by trimethylsilylation. In fact, this silylation treatment ensures completeness of reaction—and obtainment of a single main product—and conversion of all hydroxyl groups in the hormones (Kumirska et al. 2015; Ben Sghaier et al. 2017), all with adequate sensitivity and selectivity. The high volatility, low thermal degradation, fast reaction with compounds containing hydroxyl groups, and good solubility in common organic solvents of *N*,*O*-*bis*(trimethylsilyl)trifluoroacetamide led us to choose it as derivatization reagent, in combination with the catalyst trimethylchlorosilane for optimal derivatization of synthetic and natural hormones spanning a wide range of polarity (Hernando et al. 2004). Based on previous reports, BSTFA containing 1% TMCS is an excellent derivatization reagent for estrogens, androgens, and progestogens (Zuo et al. 2007; Azzouz et al. 2010; Suri et al. 2012; Albero et al. 2013; Aftafa et al. 2014; Kumirska et al. 2015; Ben Sghaier et al. 2017).

**Fig. 2:**
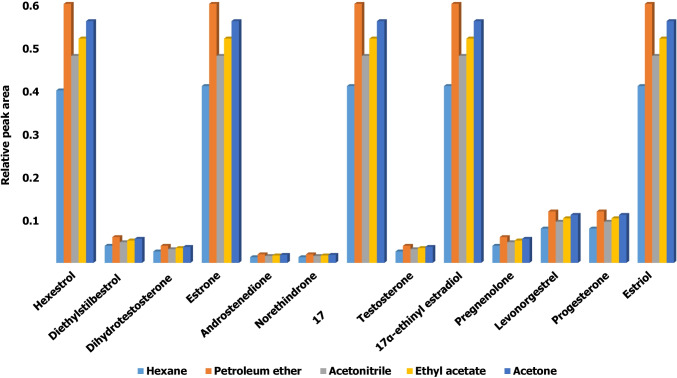
Influence of the reaction medium on the hormone derivatization reaction

Microwave irradiation has been found to reduce the time needed to derivatize analytes from several hours to a few minutes, especially with hormones (Zuo et al. 2007; Söderholm et al. 2010; Azzouz and Ballesteros 2012), provided the optimum conditions as regards solvent, irradiation time, and microwave power are established. Using an effective solvent for the sample and derivatization products is important with a view to reducing the amount of silylating reagent needed and preventing hydrolysis of the products by exposure to moisture (Bowden et al. 2009). In this work, volumes of 50 µL of individual solutions of the reagents were added to 100 µL of a solution containing a 1 µg L^−1^ concentration of each analyte in ethyl acetate, acetonitrile, acetone, petroleum ether, or *n*-hexane to identify the most suitable solvent. As can be seen from Fig. 2, the best results were obtained with petroleum ether, acetonitrile, and ethyl acetate, the first being selected on the grounds of compatibility with the stationary phase of the chromatographic column—reportedly, the BSTFA–TMCS mixture shortens column lifetime—sensitivity, and stability (Albero et al. 2013). The influence of the volumes of solvent and derivatizing reagent was examined over the range 25–100 µL, and the best results were found to be provided by 35 µL of petroleum ether and 70 µL of BSTFA + 1% TMCS.

The influence of the microwave irradiation conditions was examined by comparing the relative responses of all compounds with those obtained by using a water bath. For this purpose, a volume of 100 mL of aqueous sample containing a 100 ng L^−1^ concentration of each analyte was fed into the continuous system as described in the “Sample treatment” section. Then, the extract was evaporated to dryness under a gentle nitrogen stream and the dry residue reconstituted with 70 µL of a mixture of BSTFA + 1% TMCS and 35 µL of petroleum ether for further derivatization in the microwave oven at a variable power (100–400 W) and irradiation time (1–5 min). Using a power greater than 250 W was found to detract from the analytical signals, possibly as a result of degradation of the analytes, and so did using a power of 200 W or a time longer than 4 min. By contrast, an irradiation power below 200 W resulted in poor derivatization. A microwave setting of 200 W and an irradiation time of 4 min were thus selected. By contrast, conventional thermal derivatization required heating at 60 °C for 25 min. Using the microwave oven for silylation thus reduced the reaction time from 25 min to only 4 min, which can be especially interesting for the fast derivatization of multiclass mixtures of hormones. In any case, progesterone and androstenedione could not be derivatized because they contained no hydroxyl groups for binding to Si(CH_3_)_3_ groups in the reagent, so they had to be determined underivatized.

### Solid-phase extraction variables

Selecting an appropriate sorbent for SPE was one of the most important tasks with a view to maximizing sorption efficiency and optimizing sample treatment. Optimization tests involved passing 100 mL of an aqueous standard solution containing a 100 ng L^−1^ concentration of each analyte at pH 7, adjusted with dilute NaOH or HCl, at 5 mL min^−1^ through the sorbent column in triplicate. The sorbents included polymers (Amberlite XAD-2, Oasis HLB, and LiChrolut EN), polar materials (silica gel and Florisil), reversed phase silica sorbents containing octadecyl groups (nonpolar), and Isolute NH2, all of which were used in amounts of 80 mg to pack sorbent columns. The sorption efficiency of the sorbent materials was assessed by comparing the amounts of analytes present in 1 mL fractions of the aqueous solutions before (100% recovery) and after passage through the sorbent column (unsorbed analyte). Both fractions were collected in glass vials and evaporated to dryness under a gentle nitrogen stream for derivatization with 70 µL of BSTFA + 1% TMCS mixture and 35 µL of petroleum ether that was placed in a household microwave oven at 200 W for 4 min. Finally, 1 µL aliquots of the treated solutions were injected into the GC–MS system for analysis. As can be seen from Table 2, the highest sorption efficiency, close to 100%, for the analytes as a whole was achieved with Oasis HLB. By contrast, the efficiency of the other polymeric sorbents (LiChrolut EN, Amberlite XAD-2) and RP-C18 never exceeded 44% on average, that of Florisil and Isolute NH2 was lower than 18.2%, and that of the polar sorbent (silica gel) even lower (6.6% only).

**Table 2:** The sorption efficiency (%) of the hormones on different sorbent materials

Compound	Silica gel	LiChrolut EN	Oasis-HLB	Florisil	Amberlite XAD-2	RP-C18	IsoluteNH2
Hexestrol	30.0	55.0	98	18	22	26,5	60
Diethylstilbestrol	25,4	61.0	100	14	25,4	32	54,2
Dihydrotestosterone	5.0	33.0	97	20	35	41	1
Estrone	3,5	46,7	95	19,9	30,4	38	45,7
Androstenedione	3	28.0	95	16	30	37	0
Norethindrone	0	25,3	97	23	14,3	32,6	0
17β-estradiol	5,8	41,6	100	9,7	27,6	28,7	0
Testosterone	2.0	46.0	98	12	33	39	0
17α-ethinyl estradiol	11.0	58,9	100	27,8	46,6	36,9	76
Pregnenolone	0	22.0	97	19,5	35	11	0
Levonorgestrel	0	28,7	95	17,7	32,9	18,1	0
Progesterone	0	80,7	97	30,5	75,9	13,8	0
Estriol	0	39,4	99	3,48	17,4	34,3	0
Sorption efficiency average	**6.6**	**43.6**	**97.5**	**17.8**	**32.7**	**29.9**	**18.2**

The organic solvents used as eluents differed in polarity and included methanol, ethanol, acetone, ethyl acetate, and acetonitrile. Acetone resulted in the highest chromatographic peaks by the effect of its increased eluting efficiency, the other solvents being roughly 1.5 times less efficient. Consequently, we chose to use acetone as eluent for all hormone classes.

The influence of the amount of Oasis HLB was investigated by using various columns containing 20–120 mg of sorbent. For this purpose, a series of calibration graphs were run for each hormone and column by passing 100 mL of aqueous standard solutions containing variable concentrations (5–500 ng L^−1^) of each analyte and subsequently eluting the columns with 500 μL of acetone. Analytical signals increased with increasing amount of sorbent up to 80 mg and decreased above 90 mg owing to the need for a higher volume of eluent to ensure complete elution of the hormones. A working column packed with 80 mg of Oasis HLB was thus used in subsequent tests.

Then, the influence of eluent volumes over the range 50–800 µL was investigated by using loops of variable length in the injection valve (IV_2_ in Fig. 1). The desorption efficiency increased with increasing injected volume up to 600 µL, above which the signals for all analytes leveled off. The absence of carryover was confirmed by a second injection of 600 µL of acetone. A single injection of 600 µL of acetone therefore sufficed for complete elution of all hormones and that was the volume adopted as optimal.

Flow rates over the range 1–6 mL min^−1^ for the sample during the preconcentration step and for air (eluent carrier) during elution had no effect on analyte sorption or elution efficiency. In fact, peak areas remained constant throughout, which led us to choose 5 mL min^−1^ for both the sample and air in order to ensure a high throughput. An identical air flow rate was used to dry the sorbent column before elution.

The sample pH strongly influenced the efficiency with which the hormones were retained by the sorbent. Tests conducted over the pH range 3.0–9.0 by passing 100 mL of an aqueous standard solution containing a 100 ng L^−1^ concentration of each hormone adjusted with dilute HCl or NaOH revealed that the best results were obtained at 6.5–7.5, so neutral pH (7.0) was selected for subsequent tests. By contrast, the ionic strength of the water samples, adjusted with potassium chloride, had no effect on the analytical signal up to 1.5 M.

The breakthrough volume is important in that it is directly related to preconcentration factors, so it influences limits of detection and quantification —and hence sensitivity. The impact of this variable was assessed by passing volumes of 10–300 mL of aqueous solutions containing a 100 ng L^−1^ concentration of each analyte at pH 7 through the SPE unit. Volumes up to 200 mL resulted in no loss of analytes from the column and maximized the sorption efficiency for all analytes (∼100%); higher volumes, however, decreased the sorption efficiency by the effect of the sorbent being overloaded and/or the sample matrix being coeluted with the analytes. A sample volume of 100 mL was therefore selected that provided a concentration factor of 167 with 600 µL of solvent.

### Analytical performance

The proposed SPE–GC–MS method, depicted in Fig. 1, was used under the above-described optimum conditions to determine thirteen natural and synthetic hormones in various types of water to assess analyte detectability, linearity range, precision, and recovery. Table 1 shows the resulting figures of merit. Validation tests involved spiking 100 mL samples of ultrapure water with a few microliters of standard solutions containing all the hormones at concentrations over the range 0.04–800 ng L^−1^ that were treated as described in the “Sample treatment” section to construct calibration curves. Each calibration solution additionally contained a 500 ng L^−1^ concentration of internal standard (triphenylphosphate). The equations for the standard curves were obtained by plotting analyte-to-internal standard peak area ratios against analyte concentrations. Linearity was excellent for all analytes (correlation coefficients were all higher than 0.9981). Limits of detection (LODs), defined as the analyte concentrations providing chromatographic peaks equal to three times the regression standard deviation, *S*_*y*_/_*x*_, divided by the slope of the calibration graph, ranged from 0.01 to 0.30 ng L^−1^. LODs were also calculated as the lowest concentrations providing chromatographic signals three times higher than background noise. Tests on real samples aimed at determining LODs provided results similar to those for distilled water. The lower limit of the linear range was taken to be the limit of quantification (LOQ) and calculated as 3.3 × LOD.

The precision of the proposed method was measured in terms of reproducibility and repeatability by calculating the within- and between-day relative standard deviation (RSD), respectively, for 12 individual standard mixtures of ultrapure water containing three different concentrations of each hormone (5, 50, or 200 ng L^−1^). Analyses were performed on the same day (within-day precision) or on 7 different days (between-day precision). As can be seen from Table 1, precision was quite acceptable; thus, RSD values ranged from 3.0 to 5.5% for repeatability (within-day precision) and 4.5 to 7% for reproducibility (between-day precision). The good precision obtained can be ascribed to the use of automated SPE and an internal standard to correct chromatographic errors in terms of relative area (analyte-to-internal standard peak area ratio).

Accuracy in terms of standard deviation was checked by assessing analyte recovery from various types of water (drinking, mineral, river, swimming pool, well, and waste) that were spiked with three different concentrations of each hormone (5, 50, or 200 ng L^−1^) for analysis in triplicate (*n* = 3). Because many of the water samples studied contained some hormone, recoveries were calculated by subtracting previously quantified endogenous compounds from total contents. As can be seen from Table 3, all hormones were accurately identified; also, average recoveries were acceptable (92–103%), which testifies to the applicability of the proposed method to any type of water sample—a result, no doubt, of the highly efficient SPE unit used to pretreat samples.

**Table 3:** Average recoveries of hormones spiked to water samples^a^

Hormones	Tap		Mineral		Swimming pool		Well		River		Waste water	
	5 ng L^−1^	50 ng L^−1^	5 ng L^−1^	50 ng L^−1^	5 ng L^−1^	50 ng L^−1^	5 ng L^−1^	50 ng L^−1^	5 ng L^−1^	50 ng L^−1^	5 ng L^−1^	50 ng L^−1^
Estrogens												
Hexestrol	101 ± 5	96 ± 4	95 ± 4	103 ± 5	95 ± 5	101 ± 4	101 ± 4	98 ± 5	99 ± 5	101 ± 4	98 ± 5	100 ± 4
Diethylstilbestrol	92 ± 4	99 ± 4	100 ± 6	95 ± 4	92 ± 4	96 ± 4	96 ± 4	102 ± 4	99 ± 5	100 ± 5	101 ± 5	97 ± 4
Estrone	99 ± 4	97 ± 4	94 ± 6	103 ± 5	95 ± 4	99 ± 6	96 ± 4	98 ± 5	101 ± 4	98 ± 5	102 ± 4	95 ± 5
17β-estradiol	96 ± 4	101 ± 6	97 ± 6	94 ± 5	99 ± 6	101 ± 5	100 ± 4	99 ± 5	101 ± 5	101 ± 4	96 ± 5	102 ± 5
17α-ethinylestradiol	101 ± 5	92 ± 5	99 ± 5	92 ± 4	97 ± 6	93 ± 3	101 ± 4	101 ± 4	98 ± 4	99 ± 5	97 ± 6	102 ± 4
Estriol	99 ± 6	100 ± 5	97 ± 4	93 ± 4	103 ± 7	97 ± 5	101 ± 6	96 ± 5	102 ± 5	100 ± 5	102 ± 4	100 ± 5
Androgens												
Testosterone	100 ± 4	98 ± 4	99 ± 6	95 ± 6	100 ± 6	95 ± 4	100 ± 4	102 ± 5	97 ± 4	96 ± 4	101 ± 3	96 ± 4
Dihydrotestosterone	101 ± 4	102 ± 6	98 ± 4	100 ± 5	97 ± 4	96 ± 4	97 ± 4	92 ± 4	96 ± 4	101 ± 5	98 ± 4	100 ± 5
Androstenedione	99 ± 5	96 ± 4	100 ± 5	102 ± 4	101 ± 5	93 ± 4	92 ± 4	94 ± 4	98 ± 4	99 ± 4	99 ± 4	97 ± 4
Progestogens												
Progesterone	101 ± 5	97 ± 6	98 ± 5	95 ± 4	100 ± 4	94 ± 6	98 ± 6	97 ± 4	97 ± 4	100 ± 4	99 ± 4	98 ± 5
Norethindrone	102 ± 5	95 ± 4	102 ± 5	92 ± 4	98 ± 4	100 ± 5	93 ± 6	102 ± 5	100 ± 5	101 ± 6	99 ± 6	102 ± 5
Levonorgestrel	102 ± 5	93 ± 4	102 ± 5	97 ± 4	96 ± 5	95 ± 4	99 ± 4	97 ± 4	102 ± 5	99 ± 4	99 ± 5	100 ± 5
Others												
Pregnenolone	98 ± 4	103 ± 5	99 ± 5	94 ± 5	101 ± 5	100 ± 5	99 ± 4	94 ± 5	99 ± 4	95 ± 4	100 ± 4	97 ± 5

^a^80 mg of HLB Oasis sorbent, sample adjusted at pH 7 for all hormones; volume, 100 mL (n = 3, ± SD)

### Determination of hormones in water

The practical use of the proposed SPE–GC–MS method for determining hormones was assessed by applying it to various types of real waters samples (tap, bottled mineral, well, swimming pool, pond, river and waste) from Spain and Morocco. Volumes of 100 mL of the different types of samples were analyzed by using the method in triplicate. All samples were passed through a 0.45-µm membrane filter and adjusted to pH 7 prior to insertion into the continuous SPE system. As can be seen from Table 4, none of the hormones was present in tap or mineral water. Also, norethindrone, levonorgestrel, estriol, pregnenolone, diethylstilbestrol, and androstenedione were detected in none of the samples. On the other hand, estrone was the natural estrogen most frequently detected in all other types of water (well, pond, swimming pool, and river), but especially in Spanish and Moroccan river water (concentrations of 13 and 83 ng L^−1^, respectively), which are similar to those of coated South Florida surface water, where it was encountered at levels up to 79.5 ng L^−1^ by Ng et al. (2021). One other hormone found in many samples was dihydrotestosterone (28–64 ng L^−1^). Also, testosterone was found in Moroccan pond and Spanish and Moroccan river waters, at concentrations from 6 to 24 ng L^−1^. Ben Sghaier et al. (2017) found testosterone at levels from 5.4 to 6.5 ng L^−1^ in Belgian and French river waters (Table 5). By contrast, other hormones such as 17β-estradiol, hexestrol, 17 α-ethinylestradiol, and progesterone were found at lower levels (3.0–12 ng L^−1^) but still similar to those in surface waters from Belgium, France, or China reported by other authors (Ben Sghaier et al. 2017; Zhou et al. 2020). In the case of the samples analyzed from pond water by the proposed method, only 17β-estradiol was found in the sample from Morocco (3.6 ng L^−1^).

**Table 4:** Hormones found in water samples (± SD, ng L^−1^, n = 3) using the proposed SPE-GC–MS method

Sample	Hexestrol	Estrone	17β-estradiol	17α-ethinylestradiol	Testosterone	Dihydrotestosterone	Progesterone
Tap 1 (S)	nq^a^	nq	nq	nq	nq	nq	nq
Tap 2 (M)	nq	nq	nq	nq	nq	nq	nq
Bottled mineral 1 (S)	nq	nq	nq	nq	nq	nq	nq
Bottled mineral 2 (S)	nq	nq	nq	nq	nq	nq	nq
Well 1 (S)	nq	14 ± 1	nq	nq	nq	nq	nq
Well 2 (M)	nq	27 ± 1	3.3 ± 0.2	nq	nq	nq	nq
Swimming pool 1(S)	5.1 ± 0.3	15 ± 1	nq	nq	nq	28 ± 1	nq
Swimming pool 2 (S)	3.0 ± 0.2	43 ± 2	nq	4.0 ± 0.2	nq	31 ± 2	13 ± 1
Pond 1 (S)	nq	21 ± 1	nq	nq	nq	38 ± 2	nq
Pond 2 (M)	nq	25 ± 1	3.6 ± 0.2	nq	6.0 ± 0.3	28 ± 1	nq
River 1 (S)	nq	13 ± 1	nq	nq	17 ± 1	42 ± 2	nq
River 2 (M)	nq	83 ± 4	nq	nq	24 ± 1	64 ± 3	nq
Waste 1 (S)	10 ± 1	110 ± 10	12 ± 1	12 ± 0.1	31 ± 2	76 ± 4	24 ± 1
Waste 2 (S)	14 ± 1	93 ± 4	22 ± 1	7.0 ± 0.4	57 ± 3	45 ± 2	16 ± 1

^a^ nq: not quantified; S: Spain; M: Morocco

**Table 5:** Comparison of the proposed method with existing alternatives for the determination of hormones in aqueous samples around the world

Analytes	Samples	Countries	Pretreatment method^a^	Analytical technique^a^	Analytical features^a^	Hormone concentrations in real samples	Reference
Estrogens (and pharmaceuticals)	Surface and waste water	Poland	SPE- derivatization (BSTFA + 1% TMCS)	GC–MS GC–ECD	LOD: 2.1–4.2 ng L^–1^ RSD < 17.5% Recovery: 58–107%	8–120 ng L^–1^	Migowska et al. 2012
Estrogens	River water	Turkey	SPME	LC–MS/MS	LOD: 0.012–0.693 ng L^–1^ RSD < 10% Recovery: 87– 98%	0.10–7.53 ng L^–1^	Aftafa et al. 2014
Estrogens and progestogens	Underground, estuary, sea and waste water	Portugal	BAμE	LC–DAD	LOD: 50–100 ng L^–1^ RSD < 14% Recovery: 87–102%	300–4300 ng L^–1^	Almeida and Nogueira 2015
Estrogens, androgens, progestogens (and phenols)	River water	China	ASE–GPC–SPE-derivatization (MSTFA)	GC–MS	LOD: 0.3–0.8 ng L^–1^ RSD < 10% Recovery: 60–95%	1.2–15.9 ng L^–1^	Huang et al. 2015
Estrogens, androgens, progestogens (and other EDCs)	River water	France and Belgium	SPE- derivatization (BSTFA + 1% TMCS)	GC–MS	LOD: 0.33–3.33 ng L^–1^ Recovery: 52–71%	5.4–116 ng L^–1^	Ben Sghaier et al. 2017
Androgens, estrogens, corticosteroids and progestogens	Sea and fresh tap water	Belgium	SPE	UHPLC– HRMS	LOD: 0.06–10 ng L^–1^ RSD < 10.5% Recovery: 95–109%	0.26–39 ng L^–1^	Huysman et al. 2017
Natural and synthetic progestogens	Surface and waste water	Czechia	SPE	LC–APCI/APPI–HRPS	LOQ: 0.02–0.87 ng L^–1^ RSD < 33% Recovery: 60–140%	0.14–110 ng L^–1^	Golovko et al. 2018
Natural and synthetic progestogens	River and sewage effluents	China	SPE	LC–MS/MS	LOD: 0.008–0.12 ng L^–1^ RSD < 17% Recovery: 43–116%	0.04–38 ng L^–1^	Shen et al. 2018
Androgens, estrogens, corticosteroids and progestogens	River and waste water	Switzerland	SPE	LC–MS/MS	LOD: 0.01–40 ng L^–1^ RSD: 1–13% Recovery: 56–126%	1.0–220 ng L^–1^	Zhang and Fent 2018
Androgens, estrogens, progestogens (and bisphenol A)	Tap, surface and waste water	Canada	SPE	UHPLC–MS/MS	LOD: 0.05–1.0 ng L^–1^ RSD: 1.3–19% Recovery: 70–130%	0.80–790 ng L^–1^	Goeury et al. 2019
Androgens, estrogens and progestogens	Surface and waste water	Argentina	SPE	LC–MS/MS	LOD: 1.9–44 ng L^–1^ Recovery: 42–144%	1.9–384 ng L^–1^	González et al. 2020
Androgens, estrogens, progestogens (and other EDCs)	Swimming pool water	China	SPE	LC–MS	LOD: 0.02–0.28 ng L^–1^ RSD < 13.5% Recovery: 72–118%	0.02–78.8 ng L^–1^	Zhou et al. 2020
Natural and synthetic estrogens (and other emerging pollutants)	Surface water (river and canal)	USA	SPE	LC-HRMS	LOD: 0.2–10.5 ng L^–1^ RSD < 20% Recovery: 96–101%	5.1–285 ng L^–1^	Ng et al. 2021
Androgens, estrogens, progestogens and pregnenolone	Drinking, mineral, river, swimming pool, well and waste water	Spain and Morocco	SPE- derivatization (BSTFA + 1% TMCS)	GC–MS	LOD: 0.01– 0.30 ng L^–1^ RSD: 3.0–7.0% Recovery: 92–103%	3.0– 104 ng L^–1^	This work

^a^ASE–GPC: accelerated solvent extraction–automated gel permeation chromatography. BAμE: Bar adsorptive microextraction. BSTFA: *N*,*O*-*bis*(trimethylsilyl)trifluoroacetamide. GC–ECD: Gas chromatography with electron capture detection. GC–MS: Gas chromatography–mass spectrometry. GC–MS/MS: Gas chromatography–tandem mass spectrometry. LC–MS/MS: High performance liquid chromatography–tandem mass spectrometry. LC–DAD: High–performance liquid chromatography–diode array detection. LC–APCI/APPI–HRPS: Liquid chromatography tandem atmospheric pressure chemical ionization/atmospheric pressure photoionization with hybrid quadrupole/orbital trap mass spectrometry operated in the high resolution product scan mode. LC–HRMS: Liquid chromatography–high resolution mass spectrometry. LC–MS/MS: Liquid chromatography–tandem mass spectrometry. LOD: Limit of detection. LOQ: Limit of quantification. MSTFA: *N*-methyl-*N*-(trimethylsilyl)trifluoroacetamide. ND: Not detected. RSD: Relative standard deviation. SPE: Solid–phase extraction. SPME: Solid–phase microextraction. TMCS: Trimethylchlorosilane. UHPLC– HRMS: Ultrahigh performance liquid chromatography– high resolution mass spectrometry. UHPLC–MS/MS: Ultra–high performance liquid chromatography–tandem mass spectrometry. UWWTPs: Urban wastewater treatment plants

The wastewater samples contained various estrogens (hexestrol, estrone, 17β-estradiol, and 17α-ethinylestradiol) at concentrations from 7.0 to 110 ng L^−1^. These results are consistent with previously reported values (Table 5) such as those of Migowska et al. (2012) and Goeury et al. (2019). The androgen concentrations in wastewater were highest for dihydrotestosterone (45–76 ng L^−1^), followed by testosterone (31–57 ng L^−1^). Interestingly, these concentrations are lower than those found in Chinese wastewater (201 ng L^−1^ for dihydrotestosterone and 53.3 ng L^−1^ for testosterone; Yu et al. 2019) but higher than those in wastewater from Argentina (33 ng L^−1^ for dihydrotestosterone and 16 ng L^−1^ for testosterone; Gonzalez et al. 2020). Progesterone, at 16–24 ng L^−1^, was the only progestogen detected in Czech wastewater, these levels being considerably lower than those reported by Golovko et al. (2018) in such samples from Czechia: 0.11–110 ng L^−1^.

## Conclusion

A fast, sensitive, and chemically green method was successfully developed and validated for the simultaneous determination of three classes of natural and synthetic hormones (estrogens, androgens, and progestogens) at trace levels in environmental waters. Using offline SPE in combination with GC–MS shortens analysis times to only 15 min. The proposed method has a number of advantages such as the following:

1. Some of the studied compounds are set to be regulated by its toxicological potential in the upcoming legislation in the EU (EC 2020).
2. Using a continuous SPE system to preconcentrate the analytes reduces the amount of sorbent needed for their retention (80 mg of Oasis HLB). Also, columns can be used at least 150 times and efficient sorption maintained with a minimal volume of organic solvent (600 µL) in a closed system that avoids analytes losses and reduces the risk of sample contamination from the laboratory or the analyst. The use of these systems significantly reduces the consumption of samples, reagents, and organic solvents in compliance with the principles of Green Chemistry.
3. Using microwave radiation in combination with a silylation reagent affords fast derivatization of a multiclass hormones mixture (4 min vs 25 min with conventional methods).
4. Precision (RSD values of 3.0–7.0%), accuracy (recoveries of 92–103%), and limits of detection (0.01–0.30 ng L^−1^) are much better than those of existing alternatives (Table 5). In fact, LODs are well below the limits set on the European Watch Lists (e.g., 0.035 ng L^−1^ for 17α-ethinylestradiol and 0.4 ng L^−1^ for estrone and 17β-estradiol) (EC 2018).
5. Four estrogens (estrone, 17β-estradiol, 17α-ethinylestradiol, and hexestrol), two progestogens (testosterone, dihydrotestosterone), and one progestogen (progesterone) at concentrations ranging from 3.0 to 110 ng L^−1^ have been found in the different types of water samples analyzed. In all cases, the concentrations of hormones found in the water samples collected in Spain and Morocco are lower or similar to those found by other researchers in samples from many areas of the world as can be seen in Table 5.

In summary, the proposed method is a good choice for the routine analysis of real water samples (drinking, mineral, well, swimming pool, pond, river, and waste) for natural and synthetic hormones.

## Data Availability

Not applicable.
